# A Critical Role of the Nuclear Receptor HR3 in Regulation of Gonadotrophic Cycles of the Mosquito *Aedes aegypti*

**DOI:** 10.1371/journal.pone.0045019

**Published:** 2012-09-26

**Authors:** Daniel Mane-Padros, Josefa Cruz, Andrew Cheng, Alexander S. Raikhel

**Affiliations:** Department of Entomology and Institute of Integrative Genome Biology, University of California Riverside, Riverside, California, United States of America; Centro de Pesquisas René Rachou, Brazil

## Abstract

The orphan nuclear receptor HR3 is essential for developmental switches during insect development and metamorphosis regulated by 20-hydroxyecdysone (20E). Reproduction of female mosquitoes of the major vector of Dengue fever, *Aedes aegypti*, is cyclic because of its dependence on blood feeding. 20E is an important hormone regulating vitellogenic events in this mosquito; however, any role for HR3 in 20E-driven reproductive events has not been known. Using RNA interference (RNAi) approach, we demonstrated that Aedes HR3 plays a critical role in a timely termination of expression of the vitellogenin (*Vg*) gene encoding the major yolk protein precursor. It is also important for downregulation of the Target-of-Rapamycin pathway and activation of programmed autophagy in the *Aedes* fat body at the end of vitellogenesis. HR3 is critical in activating *betaFTZ-F1*, *EcRB* and *USPA*, the expressions of which are highly elevated at the end of vitellogenesis. RNAi depletion of *HR3* (iHR3) prior to the first gonadotrophic cycle affects a normal progression of the second gonadotrophic cycle. Most of ovaries 24 h post second blood meal from iHR3 females in the second cycle were small with follicles that were only slightly different in length from of those of resting stage. In addition, these iHR3 females laid a significantly reduced number of eggs per mosquito as compared to those of iMal and the wild type. Our results indicate an important role of HR3 in regulation of 20E-regulated developmental switches during reproductive cycles of *A. aegypti* females.

## Introduction

Female mosquitoes serve as vectors for harmful human diseases because blood feeding is required for their egg development. As a consequence disease pathogens utilize hematophagous female mosquitoes for obligatory stages of their life cycles. Reproduction of anautogenous mosquitoes is cyclic because of its dependence on blood feeding. Each reproductive cycle of an *A. aegypti* female is separated into two distinct periods: previtellogenic and vitellogenic. A newly eclosed female mosquito undergoes a post-eclosion development controlled by juvenile hormone III, during which it becomes competent for host-seeking behavior, blood digestion, and egg development [1], [2]. This phase is followed by a state-of-arrest (a reproductive diapause), which is maintained until a blood meal is taken–nutritional control via amino acid and insulin initiation of the Target-of-Rapamycin (TOR) pathway is essential for activating vitellogenesis [3]–[7]. Vitellogenesis is a key event of egg maturation, during which the fat body (FB)–which is a functional analogue of the vertebrate liver–produces massive amounts of yolk protein precursors (YPPs) to be taken up by oocytes; 20-hydroxyecdysone (20E) controls vitellogenesis [1], [2].

Molecular elucidation of the 20E genetic hierarchy has led to the identification of the ecdysone receptor as a heterodimer of two nuclear receptors–EcR and USP [8], [9]. Further studies have shown that the action of the EcR/USP is mediated by early genes–*broad (br), E74,* and *E75*–that encode transcription factors involved in regulation of 20E target genes [10]; [11]. The 20E-mediated regulatory network is further refined by the presence of genes required for setting up the stage specificity of gene activation [10]–[12].

A blood meal triggers a 20E cascade that activates *YPP* genes in the *A. aegypti* FB [1], [2]. Deciphering the 20E genomic hierarchy regulating vitellogenesis has been predominantly focused on the *Vg* gene, encoding the major YPP [1], [2]. Ecdysone response elements (EcRE) have been found in the 5′-upstream region of the *A. aegypti Vg* gene along with those of E74, E75, and BR, thus indicating that it is the target of direct and indirect regulation by 20E [13]–[16]. Differential roles of isoforms BR, E74, and E75 in governing mosquito vitellogenesis have been elucidated by means of RNAi depletion [16]–[18]. Programmed autophagy plays a significant role in FB remodeling during the termination phase, and it is essential for a developmental switch to the second gonadotrophic cycle [19].

The orphan nuclear receptor HR3 is recognized as a central regulator in 20E-driven developmental switches during insect development and metamorphosis, and is responsible for directing timely shutdown of early genes regulated by a preceding 20E peak and a sequential activation of factors by a subsequent pulse of 20E (Fig. S1) [20]–[23]. In the mosquito *A. aegypti*, vitellogenic expression of the *HR3* gene occurs before expression of the competence factor betaFTZ-F1, suggesting involvement of these factors in orchestrating stage-specific transitions during vitellogenic cycles [24], [25]. The termination of *YPP* gene expression is important so that a switch to another egg developmental cycle can be initiated. Analysis of mechanisms governing these genes is essential for understanding cyclicity of egg production in mosquitoes, which serves as a foundation for pathogen transmission. However, the HR3 involvement in regulating mosquito egg developmental cycles has not been functionally characterized.

In this study, we observed that the Aedes HR3 orthologue (AaHR3) played a critical role in a timely down regulation of *Vg* gene expression, as demonstrated by means of RNA interference (RNAi). HR3 exerted its negative regulation via direct binding to its cognate recognition sites in the *Vg* promoter. HR3 was also shown to be important for a timely shutdown of the TOR pathway and activation of programmed autophagy in the *Aedes* FB at the end of vitellogenesis. Moreover, HR3 was critical in activating betaFTZ-F1 isoforms A and B, EcRB and USPA, all of which were highly elevated at the end of vitellogenesis. RNAi depletion of HR3 prior to the first gonadotrophic cycle also affected normal progression of the second gonadotrophic cycle. Taken together, our results indicate an important role of the nuclear receptor HR3 in regulation of 20E-regulated developmental switches during reproductive cycles of females of the *A. aegypti* mosquitoes.

## Results

### Effect of RNAi Depletion of *AaHR3* on Vitellogenic Events in *Aedes aegypti* Female Mosquitoes

**Figure 1:**
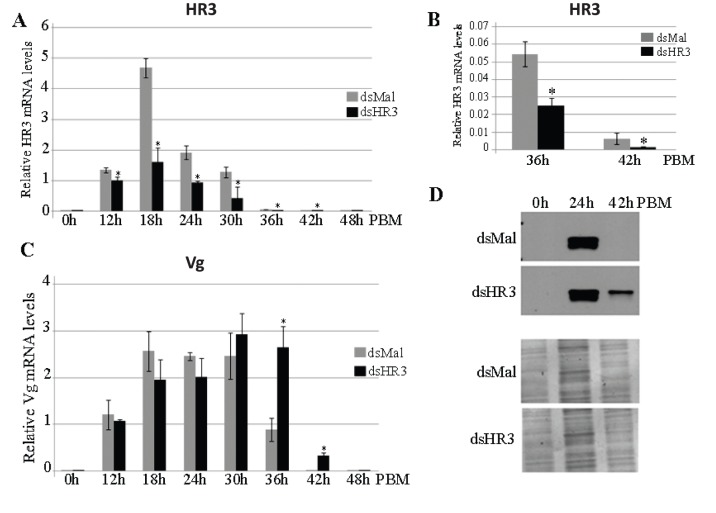
HR3-knockdown *Aedes* mosquitoes showed a delay in impaired vitellogenin (Vg) mRNA and Vg protein levels at the end of vitellogenesis in the fat body. (A–C) Female mosquitoes were injected with 1 µg dsHR3 or dsMal RNAi, as described in Materials and Methods. HR3 (A and B) and Vg (C) mRNA expression was measured using qPCR at the indicated time points after blood feeding (PBM). Point 0h represents 72h post-eclosion in female mosquitoes prior to blood feeding; this was used as a reference control. Data are expressed as fold induction relative to S7. Data (means + standard errors of the means) from three independent experiments are shown. *Indicates statistical significance <0.05. (B) Magnification scale of the 36-h and 42-h PBM time points from the same experiment shown in A to demonstrate details of the HR3 depletion at the end of the vitellogenic cycle. (C) Monitoring the Vg transcript abundance in the same experiments as in A. A high level of the Vg transcript was observed in FBs of HR3-depleted female mosquitoes at 36 h PBM showing a delayed high expression of the *Vg* gene. (D) Western blot analysis of Vg protein during several time points of the first gonadotrophic cycle in the fat body of female mosquitoes treated with dsMal or dsHR3 RNAi. A total of 0.01 fat body equivalents was loaded in each lane for SDS/PAGE. A mixture of nine monoclonal antibodies against Vg small subunit was used for Vg protein detection (upper panels). Lower panels with Comassie-stained SDS/PAGE gels used for the western blot experiment are presented as loading controls. Three independent replicates of the experiment were made.

We studied the effect of *AaHR3* depletion in the FB of *A. aegypti* female mosquitoes during vitellogenesis by means of RNAi analysis. Female mosquitoes injected with either HR3 dsRNA or control Mal dsRNA were blood fed, and FBs were dissected at different time points during the first vitellogenic cycle (between 0 h and 48 h post blood meal, PBM). 0 h represents the time prior to blood feeding and 4 days after dsRNA injections. After RNA extraction and cDNA synthesis, levels of AaHR3 and Vg mRNAs were measured using quantitative PCR (qPCR). In mosquitoes injected with dsMal, the level of AaHR3 mRNA increased at 12 h PBM and showed a sharp peak of expression at 18 h PBM. Later, this level decreased, reaching a background level by 48 h PBM (Fig. 1A and Fig. 1B). This is in agreement with previously published results for untreated female mosquitoes [24]–[26]. However, when mosquitoes were treated with dsHR3, a significant reduction in AaHR3 mRNA levels was observed, especially between 18 h and 30 h PBM (Fig. 1A). Even at 36 h and 42 h PBM, when HR3 levels were lower, a difference in HR3 expression level could be detected between dsMal and dsHR3 knockdown mosquitoes (Fig. 1B). This result shows that AaHR3 transcript was efficiently depleted in the FB by means of RNAi.

Next, we examined the effect of *AaHR3* knockdown on the expression of the *Vg* gene in the same FBs (Fig. 1C). In dsMal control female mosquitoes, the levels of Vg transcript increased during the first 12 h PBM and reached maximum levels at 18–30 h PBM; Vg mRNA level remained high until 30 h PBM and then rapidly declined, returning to a basal level by 48 h PBM. In FBs of dsHR3-treated female mosquitoes, levels of Vg mRNA were similar to those treated with dsMal until 30 h PBM. However, in the former, the level of Vg transcript remained high at 36 h PBM, the time when it normally significantly declines (Fig. 1C).

We then tested the effect of *AaHR3* RNAi depletion on the Vg protein level in FBs. Mosquitoes were collected from the same batches of female mosquitoes as for mRNA level assays. Nine FBs were analyzed at each time point–0, 24, and 42 h PBM. Vg protein levels were detected by means of western blotting using a mixture of monoclonal antibodies specific to the Vg small subunit (Fig. 1D). In FBs from dsMal control mosquitoes, Vg protein was present at the high level 24 h PBM but was not detectable at either 0 h or 42 h PBM. However, in FBs of dsHR3-treated mosquitoes, this Vg protein was still present at 42 h PBM (Fig. 1D).

To confirm the *in vivo* results with *AaHR3* RNAi depletions, we employed *in vitro* FB culture. Female mosquitoes were injected with dsHR3 or dsMal. After 4 days, FBs were dissected and incubated for 6 h in a complete culture medium supplemented with amino acids in the presence or absence of 10^−6^ M 20E (Fig. S2). The effect of *HR3* depletion on Vg mRNA expression was then determined using qPCR. The level of HR3 mRNA was elevated in FBs from dsMal-depleted mosquitoes incubated in the presence of 20E, but not in those incubated in the control medium lacking 20E (Fig. S2A). Depletion of *AaHR3* abolished such an increase in the presence of 20E (Fig. S2A). While the level of Vg mRNA did increase in FBs of dsMal-depleted mosquitoes incubated for 6 h in the presence of 20E, it was highly elevated in this tissue from dsHR3-depleted mosquitoes incubated under the same conditions (Fig. S2B). These results corroborate the *in vivo* experiments and indicate that the observed effects were due to HR3 RNAi depletion in the FB tissue. Taken together, these *in vivo* and *in vitro* experiments suggested that HR3 was important for a timely repression of *Vg* transcription in the FB during vitellogenesis.

**Figure 2:**
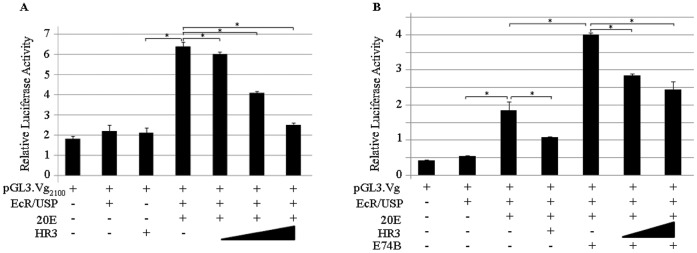
HR3 inhibits 20E-dependent activation of the *A. aegypti* Vg gene promoter in luciferase reporter assays. (A) Drosophila KC-L57-3-11 cells were transiently transfected with 300 ng of the reporter construct pGL3.Vg_2100_ (carrying 2.1 Kb of the *A. aegypti Vg* promoter) and the expression plasmids pAc5.EcR (0.2 microg) and pAc5.USP (0.2 microg), indicated as EcR/USP. The expression plasmid pAc5.HR3 (HR3) was added at 0.2, 1 or 2 microg. Expression assays were conducted in the presence (+) or absence (−) of 1 µM 20E for 36 h. 50 ng of the control expression vector pRLCMV. Renilla luciferase was co-transfected for normalization. (B) The assay was conducted in a similar manner, but with the addition of 0.2 µg of the expression plasmid pAc5.E74B (E74B). The expression plasmid pAc5.HR3 was added at 1 or 2 microg. Relative luciferase activity represents ratios of firefly luciferase to Renilla luciferase readings. Values are the mean of three replicates (± SEM). The experiment was repeated three times. *Indicates statistical significance <0.05.

To test further whether the effect of AaHR3 on *Vg* transcription was as a result of its effect on the *Vg* promoter, we performed a luciferase cell transfection assay using the reporter construct pGL3.Vg_2100_ in *Drosophila* L57-3-11 cells, which lack endogenous EcR (Fig. 2). In the absence of 20E, only basal levels of luciferase activity were observed when the reporter construct was transfected alone, together with EcR and USP expression plasmids (EcR/USP) or a HR3 expression vector. When pGL3.Vg_2100_ was co-transfected with EcR and USP expression plasmids in the presence of 10^−6^ M 20E in the culture medium, there was a 3-fold increase in luciferase activity. However, addition of increasing amounts of the AaHR3 expression vector negatively affected the EcR/USP/20E-driven activation of the reporter. The activation of the *Vg_2100_* promoter was reduced to a background level after co-transfection of the 10-fold excess of the AaHR3 expression plasmid (Fig. 2A). Luciferase assays indicated that AaHR3 inhibited the ability of the heterodimer EcR/USP to activate the *Vg* promoter in the presence of 20E.

The product of the early gene *E74B* is required for a high level 20E-dependent *Vg* gene expression (16). When the E74B expression plasmid was co-expressed together with EcR and USP (EcR/USP) expression vectors and the reporter construct pGL3.Vg_2100_ in the presence of 20E, the luciferase activity was sharply elevated (Fig. 2B). However, addition of the AaHR3 expression plasmid significantly repressed this elevation (Fig. 2B). These experiments suggested that AaHR3 directly repressed the *Vg* gene by binding to its cognate sites in the *Vg* promoter. However, because *Drosophila* L57-3-11 cells are void of endogenous EcR, but not other 20E-dependent factors, we cannot rule out another scenario under which HR3 activated inhibitory factors such as E75 or B which in turn would repress the Vg/luciferase activity.

### Effect of *HR3* Depletion on Expression of Genes Involved in the 20E Regulatory Cascade in the Fat Body of *A. aegypti* Female Mosquitoes

**Figure 3:**
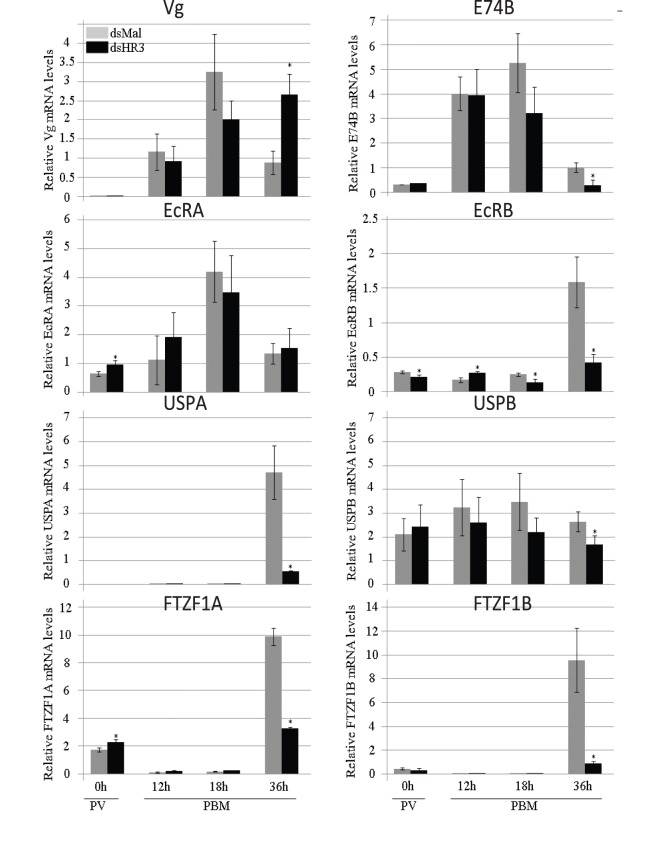
Effect of HR3 RNAi depletion on mRNA abundance levels of genes involved in 20E response in the fat body of *Aedes* female mosquitoes. Female mosquitoes were injected with 1 µg of dsHR3 or dsMal. Transcript levels of Vg, E74B, EcR-A, EcR-B, USP-A, USP-B, betaFTZF1-A, and betaFTZF1-B were quantified by means of qPCR during a time-course experiment that covered from previtellogenesis (PV) to 36 h PBM. Each time point represents the average (± SEM) of three groups of three FBs. Each sample was normalized to its internal control ribosomal protein-7 mRNA. Three independent replicates of the experiment were assayed with three different cohorts of mosquitoes. *Indicates statistical significance <0.05.

The process of vitellogenesis (governed by 20E) in the FB of *A. aegypti* females involves synchronized activation of a set of early gene products, which leads to a high level of *Vg* gene expression by 24 h PBM [16]–[18]. In addition, several 20E-induced transcription factors are involved in termination of vitellogenesis [15], [17], [18]. The product of early gene, isoform E74B, is essential for a high level of expression of the *Vg* gene in the Aedes female FB [16]. In agreement with previously reported data, we demonstrated E74B transcript levels reached maximum at about 18 h PBM, after which they declined (Fig. 3). In FBs of *HR3*-depleted mosquitoes, the E74B mRNA was at a lower level 36 h PBM than in Mal controls (Fig. 3B).

Previously, we have characterized two Aedes EcR isoforms, EcRA and EcRB, which have dissimilar profiles of expression in the FB [26], [27]. In our present study, we have shown that RNAi depletion of *AaHR3* did not affect the levels of EcRA mRNA. In contrast, AaHR3 depletion of resulted in a dramatic reduction of the EcRB transcript level at 36 h PBM when compared with dsMal control (Fig. 3). Two Aedes heterodimeric partners of EcR–USPA and USPB–are differentially expressed in the FBs during the vitellogenic cycle. USPA was highly expressed in dsMal control mosquitoes at 36 h PBM, but this elevation was completely eliminated by depletion of AaHR3. USPB that is expressed continuously throughout the vitellogenic cycle was not affected by AaHR3 depletion (Fig. 3).

The nuclear receptor betaFTZ-F1–called the competence factor–is important for maintaining 20E-regulated developmental switches during development and metamorphosis of *Drosophila melanogaster* [28]. In *A. aegypti*, this receptor is required for the onset of vitellogenesis and successful egg development during the first cycle; its expression is elevated in the previtellogenic stage and then declines during the synthesis stage of vitellogenesis. In addition, the *betaFTZ-F1* gene is expressed again at the cessation of vitellogenesis, following upregulation of the *HR3* gene, presumably in preparation for the next vitellogenic cycle [29]. More recently, it has been shown that there are two betaFTZ-F1 isoforms in *A. aegypti* [27]. In our experiments, betaFTZ-F1A was expressed at the previtellogenic stage, declined during vitellogenesis and was highly elevated at 36 h PBM, the time of termination of vitellogenesis. BetaFTZ-F1B was also highly elevated at 36 h PBM. Depletion of AaHR3 eliminated elevation of both betaFTZ-F1 isoforms at 36 h PBM when compared with the Mal control treatment, in which both isoforms had a high level of expression similar to that reported in wild-type untreated mosquitoes (Fig. 3). Thus, the above experiments with *in vivo* HR3 depletion demonstrated that a sustained high level of expression of *EcRB*, *USPA*, and two isoforms of *betaFTZ-Z1* require HR3 activity. In turn, this suggests their interaction in controlling a timely termination of vitellogenesis.

To confirm *in vivo* results with RNAi depletions, we used *in vitro* FB culture. FBs from female mosquitoes treated with dsMal or dsHR3 were incubated in a complete culture medium supplemented with amino acids in the presence or absence of 10^−6^ M 20E for 6 h or 14 h (Fig. S3). The EcRA transcript was elevated in FBs from female mosquitoes with the dsMal background after incubation with 20E for 6 h and further elevated after 14 h incubation. The level of EcRA in FBs from dsHR3-depleted females was also elevated after 6 h in the presence of 20E; however, it exhibited no further increase at 14 h of incubation. When FBs from the dsMal- or dsHR3-depleted mosquitoes were incubated for 6 h in the presence of 20E and then for 8 h in the absence of the hormone, the level of EcRA transcript was low in both depletions (Fig. S3). The situation was very different in the case of EcRB. The level of EcRB in FBs from dsMal- or dsHR3-depleted females remained at the background level, irrespective of incubation in the presence or absence of 20E. However, after FBs from dsMal or dsHR3 depleted mosquitoes were incubated for 6 h in the presence of 20E and then for 8 h in the absence of the hormone, the level of EcRB transcript dramatically increased in dsMal controls, indicating that 20E inhibited expression of *EcRB*. Under the same conditions, its transcript level remained low in FBs from HR3-depleted mosquitoes, showing that HR3 was required for its expression in the absence of 20E (Fig. S3). Interestingly, the USPA transcript exhibited a profile identical to that of EcRB in all treatments (Fig. S3). *USPB* responses to all described treatments were similar to those of *EcRA* (Fig. S3).

In FBs from dsMal or dsHR3 female mosquitoes, the betaFTZ-F1B transcript had a background level after 6 h incubation in culture medium in the absence of 20E similar to that of 0 h incubation; after incubation with 20E for 6 h in the presence of 20E, the betaFTZ-F1B transcript decreased dramatically (Fig. S3). When FBs from dsMal- or dsHR3-depleted mosquitoes were incubated for 6 h in the presence of 20E and then for 8 h in the absence of the hormone, the level of betaFTZ-F1B transcript greatly increased in FBs from dsMal female mosquitoes, but not in those from dsHR3 mosquitoes (Fig. S3). These results confirm the *in vivo* experiments and indicate that the observed effects were due to HR3 RNAi depletion in FB tissue. Taken together, these *in vivo* and *in vitro* experiments suggest that HR3 is important for a timely activation of transcripts of *EcRB*, *USPA*, and *betaFTZ-F1B* in the FB at the end of vitellogenesis in the absence of 20E.

### Programmed Autophagy is Impaired in HR3-depleted Mosquitoes at the End of the Vitellogenic Cycle

**Figure 4:**
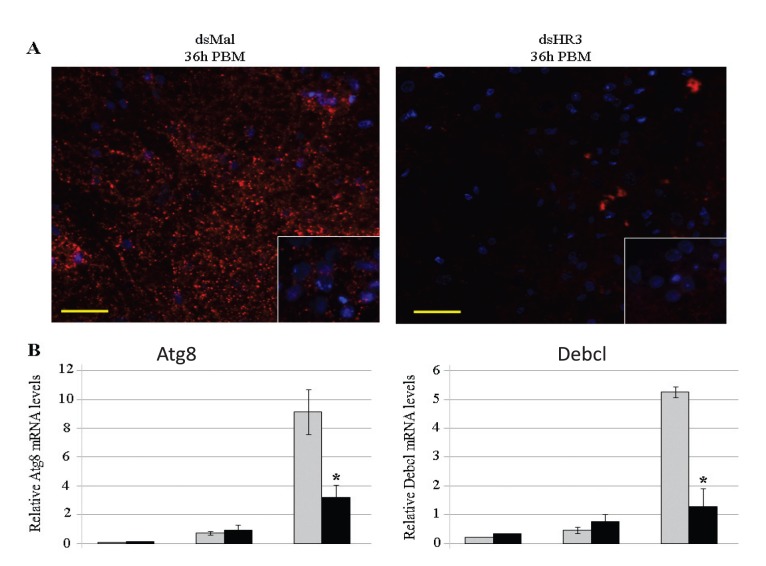
HR3 RNAi depletion impaired the programmed fat body autophagy at the end of the vitellogenic cycle. (A) Lysotracker and DAPI staining of adult female FBs, 36 h PBM, treated with 1 µg of dsMal or dsHR3 RNAi. Newly emerged female mosquitoes were injected, and five days later they received a blood meal. FBs were dissected 36 h PBM and incubated for 5 min in a solution containing 200 nM of LysoTracker Red DND-99 and 0.01 microg/microl of DAPI. The scale bar is 50 µm. (B) mRNA levels of two genes involved in fat body autophagy at the end of the first vitellogenic cycle in the fat body of Aedes female mosquitoes. qPCR was performed as described before using specific primers for *Atg8* and *Debcl* genes. Each sample was normalized to its internal control ribosomal protein-7 mRNA and three independent experiments were assayed. *Indicates statistical significance <0.05.

Autophagy is a highly conserved process of degradation and recycling of cellular components under conditions of stress, starvation and developmental transitions [30]. Programmed autophagy of the FB is required for maintaining egg maturation cycles in female Aedes mosquitoes [19]. EcR RNAi depletion demonstrated its activating role of autophagy [19]. We showed here that depletion of HR3 resulted in a dramatic decrease of transcript levels of EcRB isoform at the time of termination of vitellogenesis (Figs. 4 and S3). Thus, we tested a hypothesis that HR3 may be involved in control of autophagy at the end of vitellogenesis. In order to visualize autophagic activity in the mosquito FB, we used the fluorescent dye LysoTracker Red, a marker widely used in studies of autophagy [31]. Our previous study showed that this staining reached maximal intensity in FBs at 36 h PBM, the time of termination of vitellogenesis [19]. We examined the effect of HR3 depletion on autophagy at 36 h PBM. Fat bodies from dsMal control mosquitoes exhibited a high level of LysoTracker staining. In contrast, dsHR3 mosquito FBs showed almost no LysoTracker staining, indicating that autophagy was blocked as a result of HR3 depletion (Fig. 4A).

For further analyses, we selected *ATG8*, the gene encoding a critical initiator of vesicle expansion during autophagy [30]. The ATG8 transcript starts rising at 12 h PBM and reaches its peak at 36 h PBM [19]. In FBs of Mal control mosquitoes, ATG8 transcript level reached a peak at 36 h PBM; however, it was only slightly elevated in HR3-depleted mosquitoes (Fig. 4B). Pro-apoptotic effector Debcl has also been shown to be involved in autophagy [32]. In the female mosquito FB, a transcript level of the Debcl orthologue reaches its peak at 36 h PBM (not shown). A similar sharp increase was noted in the female mosquito FB after dsMal treatment (Fig. 4B). In dsHR3-treated mosquitoes, the Debcl mRNA level was not elevated at 36 h PBM (Fig. 4B). Taken together, these results suggested that HR3 was necessary for a proper induction of autophagy at the end of vitellogenesis in the female mosquito FB.

### Effect of HR3 Depletion on the TOR Pathway in the Fat Body at the End of the Vitellogenic Cycle of *A. aegypti*

**Figure 5:**
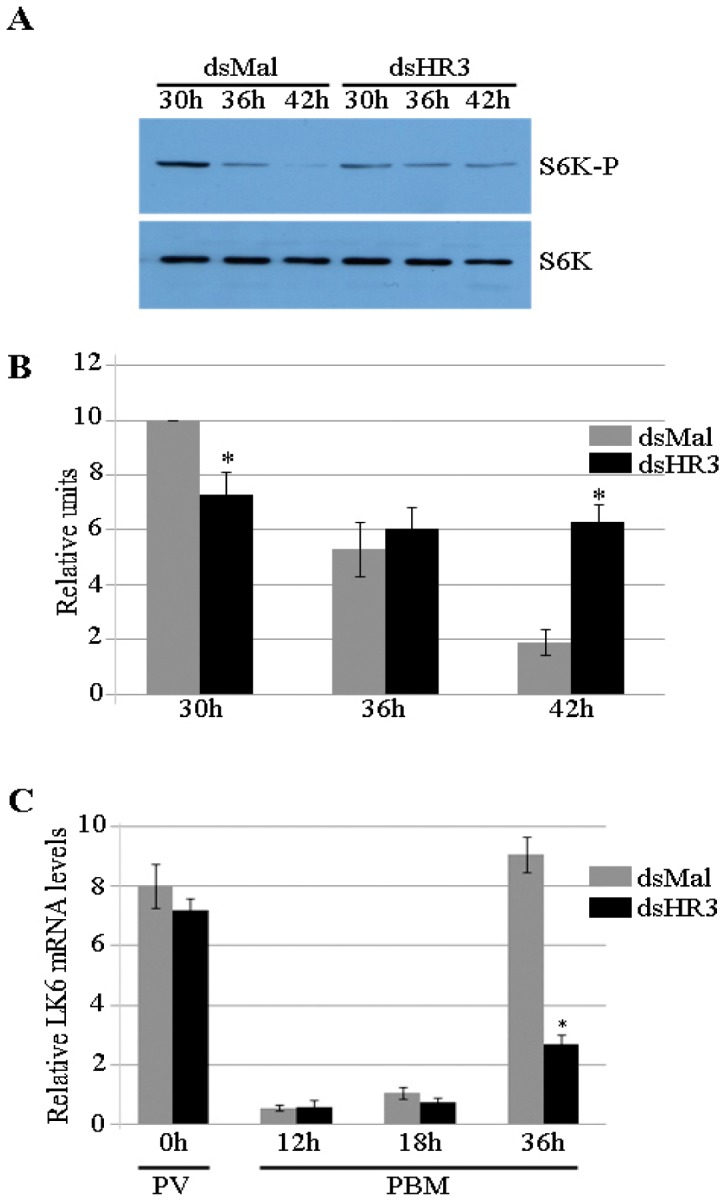
A delay in a timely shutdown of the TOR signaling in fat bodies of HR3-depleted *Aedes* female mosquitoes. (A) Western blot analysis of phospho-S6-Kinase (S6K-P) and S6-Kinase (S6K) proteins during the termination of vitellogenesis in the fat body of Aedes female mosquitoes. One fat body equivalent per lane was loaded in a SDS/PAGE gel. The experiment was done in triplicate. (B) Densitometry measurements of three independent experiments from A showing higher levels of S6K-P in dsHR3 mosquitoes. Relative units normalized to its corresponding S6K levels. *Indicates statistical significance <0.05. (C) mRNA levels of LK6-Kinase (LK6) during the first vitellogenic cycle in FBs of female mosquitoes treated with dsMal or dsHR3 RNAi. qPCR was done as previously described. *Indicates statistical significance <0.05.

Nutritional signaling mediated by the TOR pathway plays a critical role in initiation of vitellogenesis in mosquitoes [3]. TOR activity monitored by the phosphorylation status of S6K is high PBM and decreases thereafter, becoming undetectable by the time of termination of vitellogenesis [33]. As shown for *Drosophila*, autophagy is a negative regulator of TOR activity, and the autophagy initiator ATG1 inhibits TOR-dependent S6K activation via its phosphorylation [34]. Autophagy-incompetent mosquitoes exhibited a delay in termination of TOR activity [19]. Phosphorylation status of S6K in FBs of autophagy-deficient mosquitoes was elevated at 36 h and 48 h PBM, when S6K activity is normally low. Because we observed that activation of autophagy was dependent on the presence of HR3, we tested whether TOR activity was affected in FBs of HR3-incompetent mosquitoes. We analyzed the phosphorylation status of S6K in FBs of HR3-deficient mosquitoes at 30, 36 and 42 h PBM. We found an elevated phosphorylation level of S6K phosphorylation in FBs of HR3-depleted mosquitoes at 42 h PBM, when it is at a very low level in Mal controls (Figs. 5A and 5B).

LK6 kinase controls phosphorylation of eukaryotic translation initiation factor 4E and promotes normal growth and development [35]. The level of the LK6 transcript is regulated by TOR, and a low LK6 mRNA level serves as an indicator of TOR activity [35]. We performed a time-course experiment spanning from previtellogenesis to 36 h PBM and tested mRNA levels of LK6 (Fig. 5C). LK6 transcript levels were relatively high in both dsMal and dsHR3 mosquito FBs. After a blood meal, LK6 levels were low, which is in agreement with the presence of active TOR; but, at 36 h PBM, its levels exhibited a dramatic 9-fold elevation. In contrast, dsHR3-treated mosquitoes demonstrated a negligible increase of LK6 transcript at 36 h PBM, indicating again that TOR activity was maintained in HR3-depleted mosquitoes at the end of vitellogenesis (Fig. 5C).

### The Second Vitellogenic Cycle is Compromised in HR3-depleted Mosquitoes

**Figure 6:**
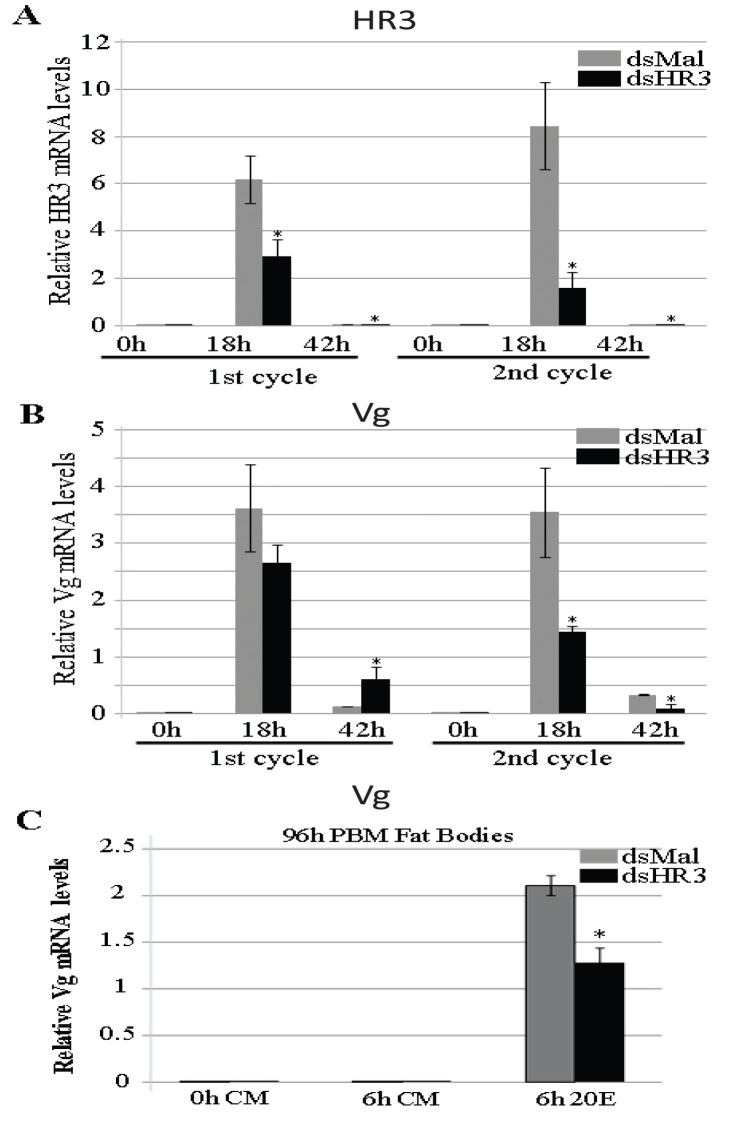
The effect of the HR3 RNAi depletion on transcript abundance of HR3 and Vg in the fat body of *Aedes* female mosquitoes during the first and second gonadotrophic cycles. Five days after RNAi injection (dsMal as a control or dsHR3), mosquitoes were given a blood meal. Mosquitoes were dissected at the indicated time points as described in Materials and Methods. To study the second vitellogenic cycle, mosquitoes received a second BM 120 h after the first. Each time point represents the average (± SEM) of three groups of three FBs. Each sample was normalized to its internal control ribosomal protein-7 mRNA. Values represent an average of three groups of three FBs. Each experiment was repeated three times (± SEM). *Indicates statistical significance <0.05. Transcript abundance of *HR3* (A) and *Vg* (B) in FBs *in vivo*. (C) Fat bodies from Aedes female mosquitoes injected with either dsMal or dsHR3 were dissected 96 h after the first blood feeding. They were incubated for 6 h in the presence (20E) or absence (CM) of 1 microM 20E. Vg relative mRNA levels were measured by means of qPCR. Each time point is the average (± SEM) of three groups of three FBs. Samples were normalized to their internal control ribosomal protein-7 mRNA. The experiment was repeated three times with different cohorts of mosquitoes. *Indicates statistical significance <0.05.

HR3 knockdown affected the proper timely termination of vitellogenesis because genes that are normally upregulated at the end of the cycle remain at very low levels (EcRB, USPA, betaFTZF1A, and betaFTZF1B). However, the level of the Vg transcript remained high in HR-depleted mosquitoes at 36 h PBM. Moreover, we showed here that FB autophagy, the process essential for maintaining vitellogenic cycles [19], was impaired in HR3-depleted mosquitoes. These results prompted us to investigate whether dsHR3 mosquitoes had a developmental disruption in the transition to the second vitellogenic cycle. To address this question, we designed an experiment in which 1-day-old previtellogenic mosquitoes were injected with dsHR3 or dsMal probes. FBs were dissected from some treated mosquitoes at several time points during the first vitellogenic cycle. Remaining mosquitoes from the same batches were fed a second blood meal after they deposited eggs, and their FBs were collected for analysis during the second vitellogenic cycle. Although the mosquitoes received only one injection of dsHR3 before the first blood meal, the HR3 transcript level remained low at 18 h and 42 h of second PBM in the second vitellogenic cycle (Fig. 6A). In FBs of dsHR3 mosquitoes, in contrast to the first vitellogenic cycle, Vg transcript levels were low at 18 h of second PBM in the second cycle and dropped to the background level at 42 h of second PBM (Fig. 6B). To confirm this effect of dsHR3 on *Vg* expression levels during the second cycle and its tissue specificity, FBs from dsHR3- and Mal-depleted mosquitoes were dissected 96 h after the first blood meal and incubated in the presence or absence of 20E for 6 h. In the presence of 20E, Vg transcript level was significantly lower in FBs from dsHR3 mosquitoes than in those treated with dsMal (Fig. 6C).

**Figure 7:**
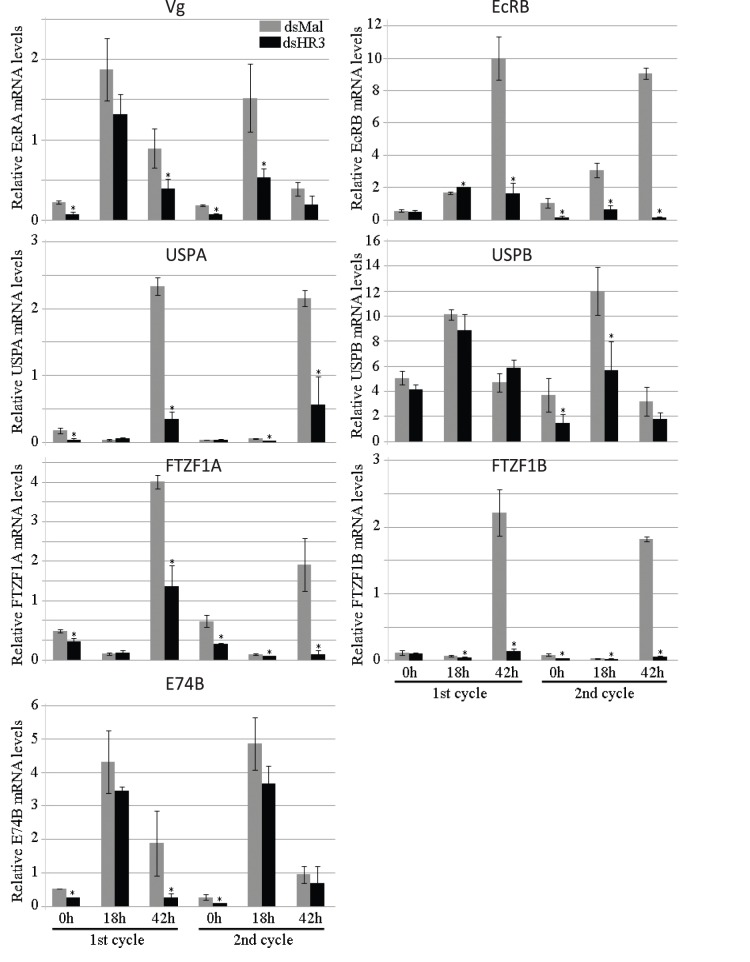
The effect of HR3 RNAi depletion on transcript levels of genes involved in the 20E response in the fat body of *Aedes* female mosquitoes during the first and the second gonadotrophic cycles. Transcript abundance of *EcRA*, *EcRB*, *USPA*, *USPB*, *betaFTZ-F1A,* and *betaFTZ-F1B* is represented. Five days after RNAi injection (dsMal as control or dsHR3), mosquitoes were given a blood meal. Mosquitoes were dissected at the indicated time points, as described in Materials and Methods. To study the second vitellogenic cycle, mosquitoes received a second blood meal 120 h after the first blood meal and egg laying. Each time point represents the average (± SEM) of three groups of three FBs. Each sample was normalized to its internal control ribosomal protein-7 mRNA. Values represent an average of three groups of three FBs. Each experiment was repeated three times (± SEM). *Indicates statistical significance <0.05.

Next, we investigated the effect of HR3 depletion on the transcript abundance of key transcription factors of the 20E cascade during the second vitellogenic cycle. In contrast to the first cycle, in which transcript levels of EcRA and USPB were not affected by HR3 depletion, they were significantly lower during the second cycle (Fig. 7). EcRB and USPA transcript levels, however, were not significantly affected by HR3 depletion during the second vitellogenic cycles of HR3-depleted mosquitoes. The betaFTZ-F1A transcript was depressed much lower at 42 h PBM of the second vitellogenic cycle than the first, but betaFTZ-F1B transcript levels were affected similarly during the first and second vitellogenic cycles in FBs of HR3-depleted mosquitoes, being dramatically decreased during both (Fig. 7). There was no difference in the effect of HR3 knockdown on E74B transcript levels in FBs during the first and second vitellogenic cycles (not shown).

**Figure 8:**
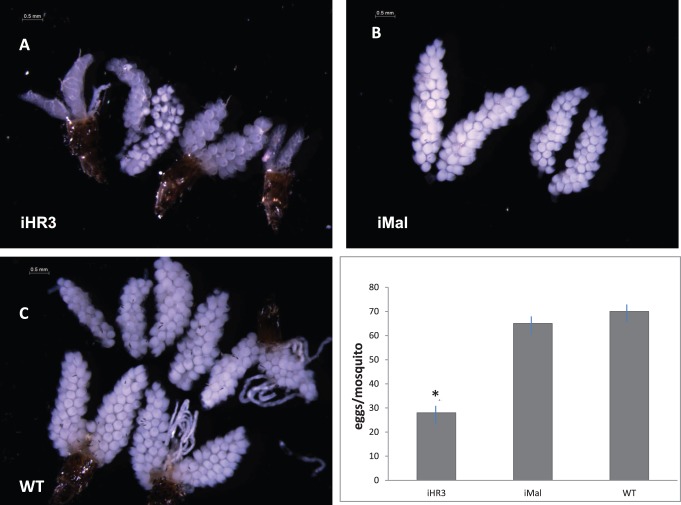
Effect of HR3 RNAi depletion on ovarian development in the second gonadotrophic cycle in *Aedes* female mosquitoes. A. Representatives of ovaries from iHR3, iMal and untreated wild type female mosquitoes at 24 h PBM. Note that in iHR3 female mosquitoes ovaries are mostly underdeveloped, or lack visible yolk mass (white). Ovaries from iMal mosquitoes were normally developed similar to those of the wild type untreated control mosquitoes. C. Number of eggs per female mosquito in the second gonadotrophic cycle. iMal - HR3 RNA depleted, iMal – dsRNA Mal-treated, and wt - wild type female mosquitoes. The experiment was repeated three times with different cohorts of mosquitoes. *Indicates statistical significance <0.05.

To investigate further HR3 RNAi depletion phenotype manifestations in the second egg maturation cycle, we evaluated the state of ovarian development in mosquitoes at 24 h PBM of the second vitellogenic cycle. Ovaries from iHR3 females were heterogeneous from small with follicles that were only slightly different in length from of those of resting stage, 100–120 µm in length to somewhat developed (Fig. 8A). Ovaries from iMal females were similar to those in untreated wild type female mosquitoes at 24 h PBM with primary follicles reaching 200–240 µm in length (Fig. 8B, 8C). In addition, these iHR3 females laid a significantly reduced number of eggs per mosquito as compared to those of iMal and the wild type (Fig. 8C).

## Discussion

**Figure 9:**
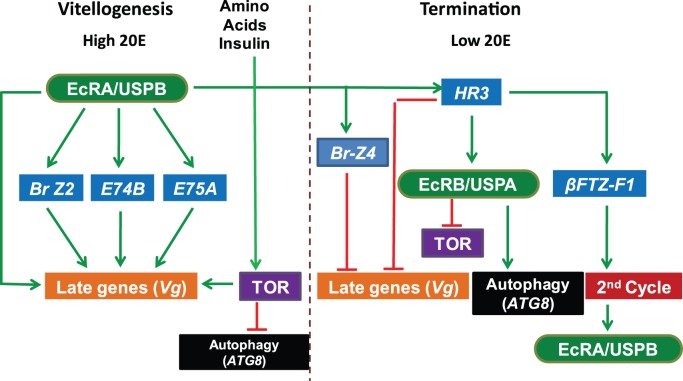
A schematic representation of the 20E regulated events during the first vitellogenic cycle in the fat body of the mosquito *A. aegypti*. After a blood meal activation, a high level of 20E acts via EcRA/USPB heterodimer activating early genes, BrZ2, E74B and E75A, which synergistically activate late genes such as *Vg* [15]–[18]. TOR, activated by amino acids and insulin, is essential for activating late genes [3], [6]. TOR is also involved in inhibition of the programmed autophagy during vitellogenesis [19]. HR3 is inhibited by E75A [18], but activated by EcRA/USPB ensuring its timely expression. At the termination time, lowering of the 20E titer results in repressive action of BriZ1 (not shown) and BrZ4 on late genes such as Vg [15], [17]. In this study, we have shown that HR3 is involved in inhibition of late target genes expression (*Vg*). On the other hand, HR3 activates the EcRB/USPA heterodimer. EcR has been shown to repress TOR and activate autophagy [19]. We postulate that it is the EcRB/USPA heterodimer that is responsible for these functions mediating action of HR3; however, this link requires additional confirmation. HR3 acts as an activator of betaFTZ-F1 that in turn is essential for maintaining cyclicity of egg development. The green ovals – EcR/USP 20E heterodimeric receptor; blue boxes – genes that encode 20E regulated transcription factors (early genes); purple boxes – Target-of Rapamycin; black boxes – autophagy; orange boxes – late target genes. Green arrows depict activating effects and red lines - repressive effects. The basics of the scheme were adapted from [46]for comparison reasons.

The orphan nuclear receptor HR3, a key regulator of 20E-mediated reprogramming in insect metamorphosis [20]–[23], [36], [37], is also expressed in the adult female mosquito *A. aegypti*. In the female mosquito, its transcript is highly elevated during the larval-pupal transition and during the peak of vitellogenesis [24], [25], [27]. Here, we showed that the HR3 transcript level is maximal at 18 h PBM of each sequential gonadotrophic cycle, coinciding with 20E peaks. 20E is required for expression of HR3 in the mosquito FB [24], [26], [27]. Experiments with *HR3* RNAi depletions have demonstrated that HR3 has multiple targets that are essential for both a programmed termination of the first vitellogenic cycle and entry into the second (Fig. 9).

A timely shutdown of expression of *ypp* genes in the FB of vitellogenic female mosquitoes, which normally occurs by 42 h PBM, is a crucial part of the termination phase. Using the *Vg* gene as the readout for vitellogenesis, we have shown that HR3 is obligatory for an appropriate cessation of expression of this major *ypp* gene. In HR3 RNAi-depleted female mosquitoes, the *Vg* transcript was abnormally elevated at 36 h PBM and still present at 42 h PBM when compared with the iMal control. Vg protein was also detected in the FBs of HR3-depleted female mosquitoes at 42 h PBM, a time at which it is normally absent (Fig. 1). The *in vivo* RNAi technique in mosquitoes is systemic; thus, *in vitro* experiments are essential in establishing whether an observed response is due to a direct effect of gene depletion in a tissue of interest rather than as a result of hindrance of a regulatory network in other organs or tissues. When FBs from HR3-depleted female mosquitoes were incubated with 20E *in vitro*, the level of the *Vg* transcript was highly elevated relative to the iMal control, confirming the *in vivo* tests (Fig. S2). The repressive function of HR3 on the *Vg* gene was confirmed by experiments utilizing the cell transfection culture assay, in which HR3 inhibited the 20E-dependent activation of the *Vg* gene reporter mediated by EcR/USP in a dose-dependent manner (Fig. 2A). Moreover, in these experiments HR3 blocked an activating function of E74B (Fig. 2B), which functions synergistically with EcR/USP in elevation of the *Vg* expression [16]. Taken together, these experiments show that HR3 is essential for repressing the *Vg* gene expression in a coordinated manner at the end of vitellogenesis. It also suggests that the 20E early gene regulatory circuitry needed for maintaining the *Vg* gene expression remains intact in the absence of HR3.

During *Drosophila* embryogenesis and metamorphosis, HR3 represses the early genes *E74A* and *E75A* of the preceding 20E-regulated cycle, permitting a developmental switch (Fig. S1) [23], [36]. However, expression of the 20E early gene regulatory circuitry, including *EcRA*, *USPB,* and *E74B*, was not affected by the HR3 depletion in FBs of vitellogenic female mosquitoes. This was confirmed by both *in vivo* and *in vitro* experiments (Figs. 4 and S3), implying that HR3 is not involved in a feedback regulation of these early genes. In contrast, our experiments indicated that HR3. Ruaud et al. [23] suggested that HR3 exerts additional functions independently of its downstream partner, betaFTZ-F1, in the Drosophila embryo. Our study has established that HR3 serves several roles essential for the termination of the vitellogenic cycle in the female mosquito. Programmed autophagy is important for FB remodeling at the end of vitellogenesis and, ultimately, for a normal progression to the next gonadotrophic cycle [19]. In this study, we have shown that HR3 is required to maintain FB autophagy, and HR3 RNAi depletion results in autophagy deficiency at 36 h PBM, when it is normally at its peak (Fig. 4A). A similar phenotype was obtained by RNAi depletion of several autophagic genes (*Atg*), including *Atg8* [19]. HR3 depletion has led to inhibition of expression of *Atg8* and *Debcl* genes (Fig. 4B), showing that HR3 is essential for activation of these key autophagic genes.

HR3 depletion resulted in a prolonged TOR activation through 42 h PBM, as could be judged from the elevated phosphorylation level of S6K (Fig. 5). We have previously demonstrated that RNAi depletion of *Atg1* alone or in a combination with either *Atg8* or *Atg6* resulted in a similar phenotype; that is, a delayed elevation of phosphorylation of S6K [19]. A link between TOR signaling and autophagy has been demonstrated in *Drosophila* larvae [29], [34]. It has been reported that Atg1, an autophagy regulator, inhibits TOR signaling and cell growth by negatively regulating S6K [38]. In this study, we have deciphered a novel function of HR3 in regulating a negative feedback loop between TOR and autophagy in the FB of vitellogenic female mosquitoes (Fig. 9). However, it remains to be established how universal this HR3 function is in 20E-regulated developmental shifts in insects.

20E signaling plays a significant part in activation of programmed developmental autophagy [39], [40]. Ectopic overexpression of *EcR* in the *Drosophila* FB induces autophagy, while expression of a dominant-negative *EcR* in this tissue of the mid third instar larvae results in a reduction of autophagy [41]. Autophagy as well as expression of *Atg1* and *Atg8* genes have been shown to be inhibited after *EcR* RNAi depletion, demonstrating that the 20E/EcR pathway is involved in regulation of *Atg* genes in the mosquito FB during termination of vitellogenesis [19]. Our *in vivo* and *in vitro* experiments demonstrated that *HR3* depletion eliminated a characteristic FB elevation of EcRB and USPA transcripts at the termination of vitellogenesis, suggesting that these isoforms likely mediate HR action in activation of programmed autophagy in this tissue (Fig. 3 and S3). However, a direct involvement of EcRB and USPA in termination of autophagy remains to be established.

During Drosophila metamorphosis and embryogenesis, HR3 acts as a reprogramming regulator by activating expression of the competence factor betaFTZ-F1. In the late third instar larva, a high titer of 20E mediated by EcR/USP activates the early genes *E74A*, *E75A*, and *Br*, products of which are responsible for activation of target genes and underlying biological responses. At the same time, EcR/USP activates *HR3* expression; in turn, HR3 inhibits *E74A*, *E75A* and *Br*, and activates *betaFtz-F1*. Activation of *betaFtz-F1* requires a low titer of 20E and occurs in mid-prepupa (Fig. S1). BetaFtz-F1 provides competence for the early genes to be reactivated by 20E in the late prepupal stage [23], [28], [36]. In the mosquito, overlapping expression of *HR3* and *betaFtz-F1* has been observed during egg maturation cycles: *HR3* is expressed during late pupal–early adult development that is followed by the elevation of the *betaFtz-F1* transcript; then the *HR3* transcript is enhanced again at 18–24 h PBM, followed by another rise in *betaFtz-F1* at the end of vitellogenesis [24], [25]. *In vitro* FB culture experiments have shown that *HR3* is activated by 20E, while *betaFtz-*F1 is inhibited; this is in agreement with observed *in vivo* expression patterns of these nuclear receptors [25], [26]. BetaFtz-F1 is essential for the mosquito FB competence to 20E response, which is accomplished by betaFtz-F1 recruitment of the p160/SRC coactivator, FISC, to EcR/USP [29], [42]. Our present study has demonstrated that HR3 is a critical factor in the orchestration of developmental shifts between vitellogenic cycles in the mosquito. *HR3* RNAi depletion resulted in a time-specific elimination of expression of *betaFTZ-F1* isoforms A and B in the mosquito FB at the termination time 36 h PBM (Fig. 3). Moreover, activation of *betaFTZ-F1B* was prevented in FBs from *HR3*-depleted mosquitoes incubated under the 20E presence/absence regimen, in contrast to the iMal control, in which the *betaFTZ-F1B* transcript was highly elevated (Fig. S3). Interestingly, RNAi depletions of *Atg* genes also inhibited elevation in *betaFtz-F1A* expression in FBs at 36 and 48 h PBM; however, the precise mechanism of autophagy involvement in activating *betaFtz-F1* in the remodeling mosquito FB is unclear [19].

Previously, we have shown that, similar to what is observed in Drosophila [43], there are three isoforms of the E75 nuclear receptor in the mosquito *A. aegypti*–E75A, E75B, and E75C [18], [44]. In Drosophila, E75B, the isoform that contains only one of the two zinc fingers making it incapable of binding DNA, is a HR3 heterodimer partner playing a critical role in the 20E-dependent developmental shifts [21], [22]. In *Manduca sexta*, E75A suppresses the *HR3* expression via its direct binding to its cognate response element [37]. RNAi depletion analysis has shown that all three Aedes E75 isoforms are important for maintaining a coordinated timely *HR3* expression in the female mosquito FB during vitellogenesis, and their depletions lead to elevated misexpression of the *HR3* gene [18]. Although RNAi depletion of *E75A* and *E75C* affected expression of the *Vg* gene, their action has been shown to be indirect [18]. Considering our present data on a direct repressive action of HR3 on the *Vg* gene expression, it is likely that this E75 action is mediated by HR3.

Multiple effects of HR3 set a stage for a coordinated transition from the first vitellogenic cycle to the second. We show here that HR3 is required for a timely repression of *Vg* gene expression (used as readout for a vitellogenic state) and the TOR activity as well as activation of programmed autophagy and the late regulatory genes, EcRB, USPA, and the betaFTZ-F1 isoforms, at the termination of vitellogenesis. In HR3-depleted mosquitoes, the *Vg* transcript remained high at the end of the first cycle, but it was low at the same time points of the second cycle (Fig. 6). This suggests that HR3 depletion affects factors essential for the 20E regulatory hierarchy in the second cycle. Indeed, *HR3* depletion decreased the transcript abundance of EcRA and USPB during the second vitellogenic cycle but not during the first (Fig. 7). It is likely that EcRB and USPA are involved in regulation of major vitellogenic events, including the maintenance of *Vg* gene expression, and that HR3 is a key factor mediating developmental switches during gonadotrophic cycles in the mosquito female. A final proof of the requirement of HR3 for coordinating the transition for the second cycle was presented by direct observations of phenotypes of iHR3 depleted females mosquitoes, which failed to normally develop ovaries and deposit a typical clutch of eggs in the second cycle. This work represents a significant advance in our understanding of the 20E-regulatory network and the nuclear receptor HR3 in governing developmental switches during mosquito egg development.

## Materials and Methods

### Animal Rearing

Mosquitoes of the Rockefeller/UGAL strain of *A. aegypti* were raised as described previously [5]. Adult females were blood fed on anesthetized white rats. All procedures for using vertebrate animals were approved by the University of California Riverside Institutional Animal Care and Use Committee (#A20100016; 05/27/2010). All dissections were performed in *A. aegypti* physiological saline (APS) at room temperature [5].

### Synthesis of Double-stranded RNA and Microinjections

Double-stranded RNA (dsRNA) synthesis was performed as described previously [18]. The following primers were used to synthesize dsRNA for RNAi knockdown of AaHR3:

5′-TAATACGACTCACTATAGGGATCGAGTTCGCCAAGCTGATA-3′.

5′-TAATACGACTCACTATAGGGATCGAGAAGAGCTCCTTGTA-3′.

These primers gave a 545-bp amplicon targeting the hinge/LBD region of HR3; underlined is the HR3 sequence. The 5′-end is the T7 sequence used for synthesis of dsRNA.

The PCR products were used as templates for dsRNA synthesis, whereas the dsRNA control was generated using the plasmid LITMUS 28iMal, containing a non-functional portion of the *Escherichia coli malE* gene. dsRNA was produced by *in vitro* transcription using the Hiscribe RNAi transcription kit (New England Biolabs, Beverly, MA). Approximately 1 microg of dsRNA in 0.2 µl of H_2_O was injected into the thorax of CO_2_-anesthetized female mosquitoes, 2 days after adult emergence, using A Picospritzer II (General Valve, Fairfield, NJ). The FBs were collected at different times for RNA analysis or *in vitro* tissue culture experiments.

### In Vitro Fat Body Culture

*In vitro* FB culture experiments were performed as described elsewhere (5). Every experiment was repeated three times and every treatment was assayed in triplicate. When needed, 20E (Sigma) was dissolved in ethanol and used at a final concentration of 1×10^−6^ M. Controls received the same volume of ethanol instead of hormone.

### RNA Extraction, Reverse Transcription, and Real-time PCR

Fat bodies that were adhered to the abdominal wall (hereafter referred to as FBs) were homogenized with a motor-driven pellet pestle mixer and lysed by Trizol reagent (Invitrogen). RNA was isolated according to the manufacturer’s protocol. Contaminating genomic DNA was removed by treatment with RNase-free DNase I (Invitrogen). A 1-microg aliquot of total RNA was reverse transcribed using a SuperScriptII reverse transcriptase kit (Invitrogen) in a 20-microl reaction mixture. cDNA levels in the different samples were quantified by means of real-time PCR (qPCR) using the iCycler iQ system (Bio-Rad). Reactions were performed in 96-well plates using a QuantiTect SYBR PCR kit (Qiagen). Data were collected with the iCycler IQ Real Time Detection System Software V3.0 for Windows. For all experiments, data were normalized against the internal control S7 ribosomal protein mRNA. A list of all primers used is available in Table S1. Statistically significant differences between samples were calculated using an unpaired Student’s *t*-test with unequal variance (Graphpad 5.0). All quantitative data were reported as mean+/−SEM.

### Lysotracker and DAPI Staining

Lysotracker staining of Aedes FB was performed as previously described [19]. FBs from female mosquitoes 36 h PBM injected with HR3 or Mal dsRNAs were dissected in APS. They were placed in an APS solution containing 200 nM of LysoTracker Red DND-99 (L7528 Invitrogen) and 0.01 microg/microl DAPI (Invitrogen), and were incubated at room temperature for 5–10 min. FBs were then mounted on glass slides and lysotracker staining was visualized under a Zeiss, AxioObserver A1 microscope.

### Cell Culture and Transient Transfection Assays

The coding region of HR3 was cloned into the pAc5·1/V5/HisA expression vector (Invitrogen). Other plasmids used in these experiments (pGL3.Vg2100, pAc5.AaEcR, pAc5.AaUSP and pAc5.74B) have been described previously [15], [16]. Transient transfection experiments were carried out in Drosophila KC-L57-3-11 cells. Cells were kept at 28°C in Schneider Drosophila medium and supplemented with 5% fetal bovine serum. Cell transfections were carried out using CellFECTIN (Invitrogen), following the protocol described previously [15]. Briefly, 200 ng of the reporter construct pGL3.Vg_2100_, 200 ng of pAc5.EcR, 200 ng of pAc5.USP and increasing amounts of pAc5.HR3 (0.2, 1 or 2 microg) were transfected together with 50 ng of the control expression vector pRLCMV.Renilla luciferase (Promega). 200 ng of pAc5.74B was added to the above mixture in experiments testing the effect of HR3 in the presence of E74B. The expression vectorpAc5·1/V5/HisA was added so that each well received the same amount of total DNA. The cells were incubated with the transfection cocktail for 6h, and then it was replaced with fresh growth media. Cells were lysed 36 h later in 100 µl of passive lysis buffer (Promega). Luciferase activities were measured using the Dual Luciferase Kit (Promega) and a Turner Biosystems Luminometer. Relative luciferase activity was calculated by normalization of the firefly luciferase activity against renilla luciferase activity. Treatments were made in triplicate, and transfections were repeated three times.

### Antibodies and Immunoblot

Protein analysis of Vg and TOR signaling was done as described previously [5], [7], [33]. FBs from blood-fed female mosquitoes were homogenized in lysis buffer (50 mM Tris HCl, pH 7.4, 1% NP-40, 0.25% sodium deoxycholate, 150 mM NaCl, 1 mM EDTA, 1 mM PMSF, 1× phosphatase inhibitor from Sigma cat # P2850, and 1× protease inhibitor from Sigma cat # P8340) and run on Tris-Glycine gels (Invitrogen) before being transferred to PVDF membranes. For detection of Vg protein, a mixture of Vg monoclonal antibodies [45] was used at the 1∶5000 dilution; this was followed by the secondary anti-mouse-HRP (cat # sc-2005 Santa Cruz) at the 1∶2000 dilution. For detection of phosphorylated S6K, the anti-human S6K-P antibody, recognizing a conserved Tyr 388 (Upstate Millipore, cat # 07-018), was used at the 1∶200 dilution, followed by the secondary anti-rabbit-HRP (cat # 7074 Cell Signaling) at the 1∶1000 dilution. S6K protein was used as a loading control and, for its detection, we used the polyclonal antibody against human S6K from Santa Cruz (cat # sc-230) at the 1∶100 dilution and the secondary anti-rabbit-HRP at the 1∶1000 dilution, as above.

## Supporting Information

Figure S1 **A schematic representation of the 20E-triggered regulatory interactions at the onset of** ***Drosophila*** **metamorphosis.** The green ovals – EcR/USP 20E heterodimeric receptor; blue boxes – genes that encode 20E regulated transcription factors (early genes); orange boxes – late target genes. Green arrows represent activating effects and red lines represent repressive effects. From [46] with permission.
(PDF)

Figure S2 **Effect of HR3 RNAi depletion on Vg and AaHR3 mRNA levels in the** ***in vitro*** **fat body tissue culture.** Three days after dsRNA injection to *Aedes* female mosquitoes, their FBs were dissected and incubated for 6 h in the presence (20E) or absence (CM) of 1 microM 20E. HR3 (A) and Vg (B) relative mRNA levels were measured by means of qPCR. Each time point is the average (± SEM) of three groups of three FBs. Samples were normalized to their internal control ribosomal protein-7 mRNA. The experiment was repeated three times with different cohorts of mosquitoes. *Indicates statistical significance <0.05.
(PDF)

Figure S3 **Effect of dsHR3 RNAi on mRNA transcript levels of genes involved in 20E response in the** ***in vitro*** **fat body tissue culture.** Mosquitoes were injected with 1 µg of dsHR3 or dsMal RNAi. Three days later, FBs were dissected and incubated in a complete culture medium in the presence (20E) or absence (CM) of 1 microM 20E for 6 h or 14 h. In another experiment, FBs from similarly treated female mosquitoes were incubated in the 20E-containg medium for 6h, followed by 8 h incubation in the media (CM) without the hormone. Transcript levels of Vg, HR3, EcR-A, EcR-B, USP-A, USP-B, and betaFTZF1-B mRNAs were measured by means of qPCR. Each time point represents the average (± SEM) of three groups of three FBs. Each sample was normalized to its internal control ribosomal protein-7 mRNA. Each experiment was repeated three times. *Indicates statistical significance <0.05.
(PDF)

Table S1 (DOCX)
