# Supplementary figures and images for: A Critical Role of the Nuclear Receptor HR3 in Regulation of Gonadotrophic Cycles of the Mosquito Aedes aegypti
Source: PLoS One. 2012 Sep 26;7(9):e45019. doi: 10.1371/journal.pone.0045019 (PMC3458863; doi:10.1371/journal.pone.0045019)

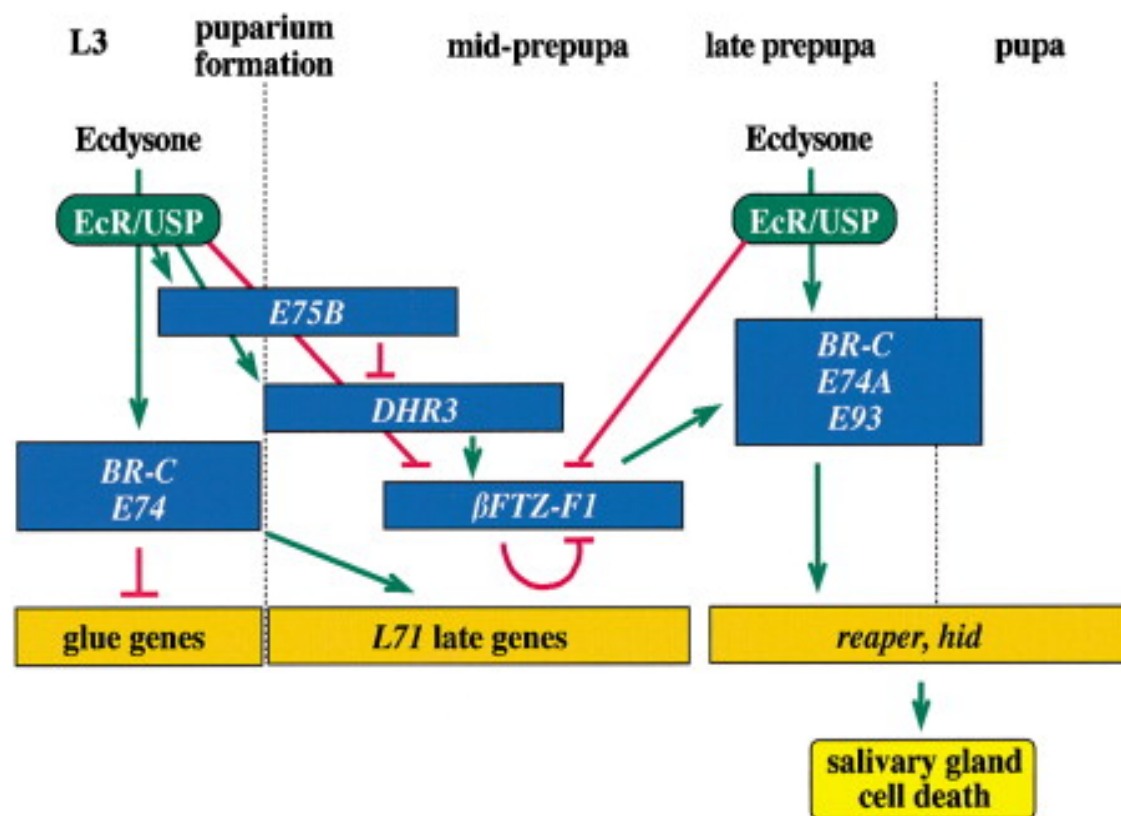

Supplement: Figure S1 — A schematic representation of the 20E-triggered regulatory interactions at the onset of Drosophila metamorphosis. The green ovals – EcR/USP 20E heterodimeric receptor; blue boxes – genes that encode 20E regulated transcription factors (early genes); orange boxes – late target genes. Green arrows represent activating effects and red lines represent repressive effects. From [46] with permission. (PDF) [file pone.0045019.s001.pdf]

# HR3

A

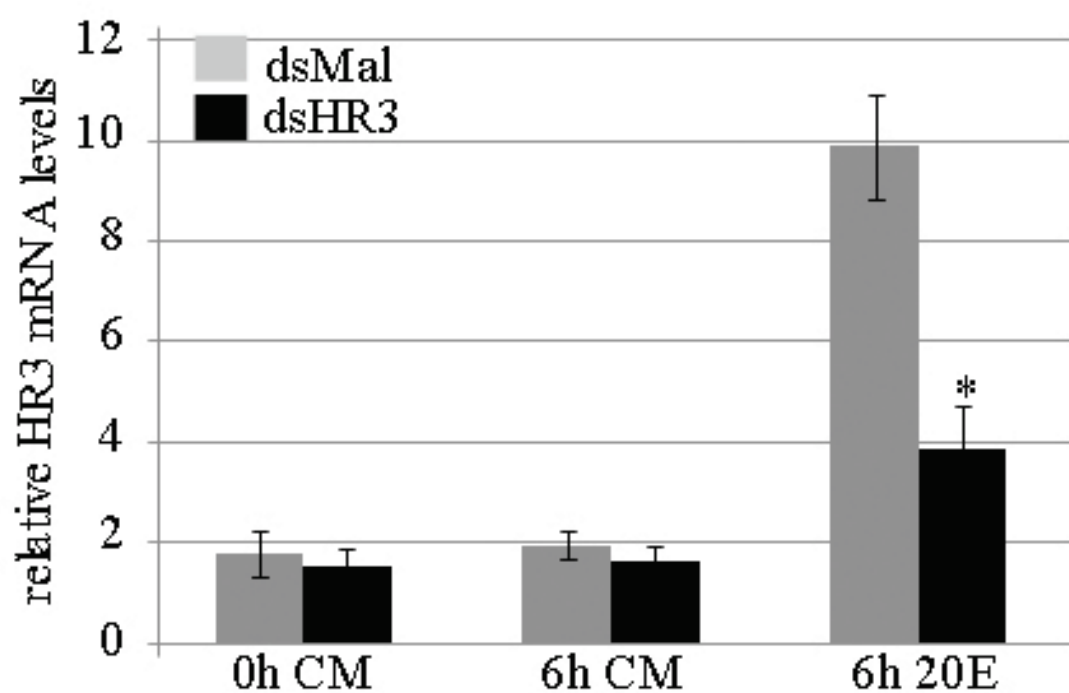

# Vg

B

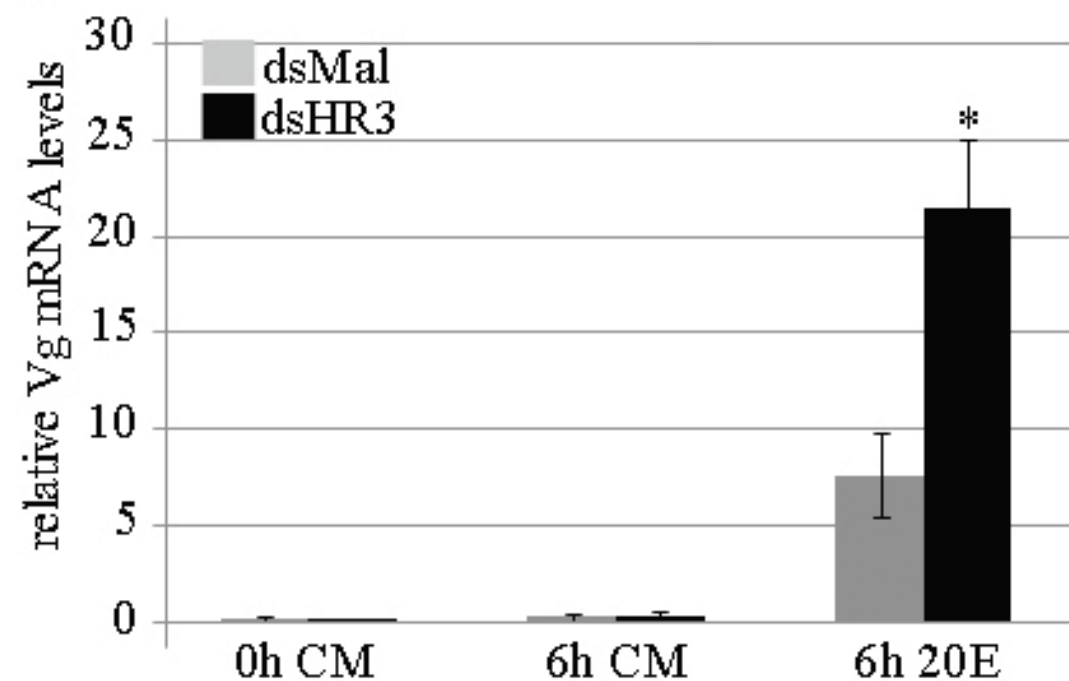

Supplement: Figure S2 — Effect of HR3 RNAi depletion on Vg and AaHR3 mRNA levels in the in vitro fat body tissue culture. Three days after dsRNA injection to Aedes female mosquitoes, their FBs were dissected and incubated for 6 h in the presence (20E) or absence (CM) of 1 microM 20E. HR3 (A) and Vg (B) relative mRNA levels were measured by means of qPCR. Each time point is the average (± SEM) of three groups of three FBs. Samples were normalized to their internal control ribosomal protein-7 mRNA. The experiment was repeated three times with different cohorts of mosquitoes. *Indicates statistical significance <0.05. (PDF) [file pone.0045019.s002.pdf]

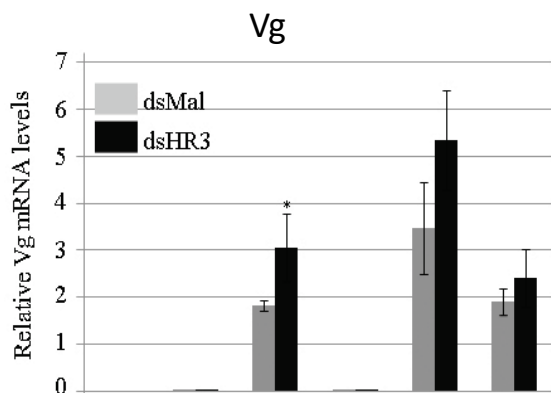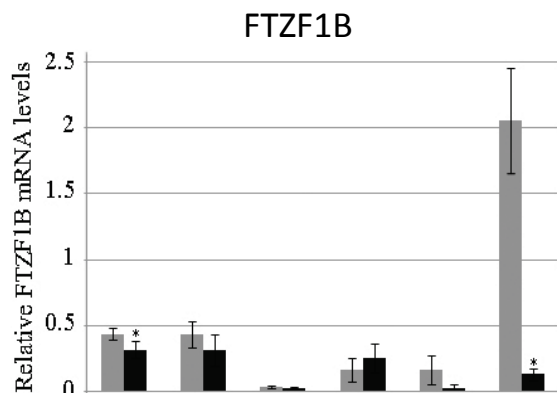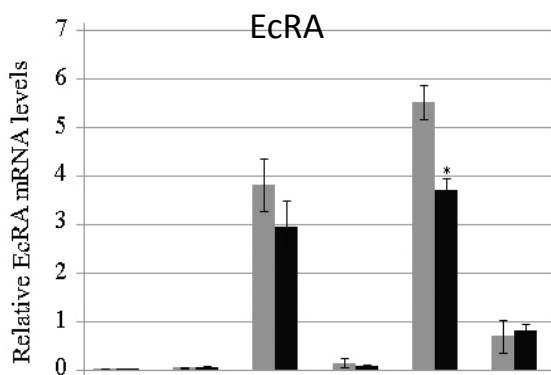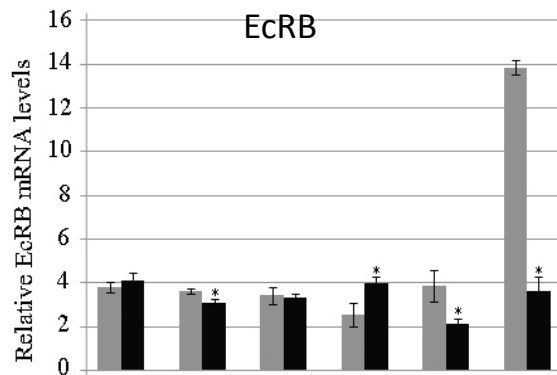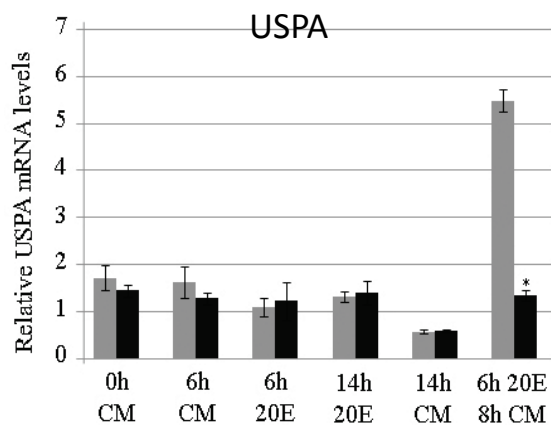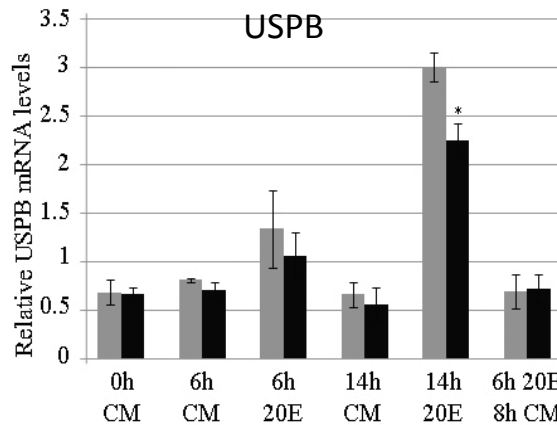

Supplement: Figure S3 — Effect of dsHR3 RNAi on mRNA transcript levels of genes involved in 20E response in the in vitro fat body tissue culture. Mosquitoes were injected with 1 µg of dsHR3 or dsMal RNAi. Three days later, FBs were dissected and incubated in a complete culture medium in the presence (20E) or absence (CM) of 1 microM 20E for 6 h or 14 h. In another experiment, FBs from similarly treated female mosquitoes were incubated in the 20E-containg medium for 6h, followed by 8 h incubation in the media (CM) without the hormone. Transcript levels of Vg, HR3, EcR-A, EcR-B, USP-A, USP-B, and betaFTZF1-B mRNAs were measured by means of qPCR. Each time point represents the average (± SEM) of three groups of three FBs. Each sample was normalized to its internal control ribosomal protein-7 mRNA. Each experiment was repeated three times. *Indicates statistical significance <0.05. (PDF) [file pone.0045019.s003.pdf]
