# Supplementary material for: A Critical Role of the Nuclear Receptor HR3 in Regulation of Gonadotrophic Cycles of the Mosquito Aedes aegypti
Source: PLoS One. 2012 Sep 26;7(9):e45019. doi: 10.1371/journal.pone.0045019 (PMC3458863; doi:10.1371/journal.pone.0045019)
Supplement: Table S1 — (DOCX) [file pone.0045019.s004.docx]

| Primer | Sequence |
| --- | --- |
| Vg forward | 5′-ATGCACCGTCTGCCATC-3’ |
| Vg reverse | 5′-GTTCGTAGTTGGAAAGCTCG-3’ |
| S7 forward | 5′-TCAGTGTACAAGAAGCTGACCGGA-3’ |
| S7 reverse | 5′-TTCCGCGCGCGCTCACTTATTAGATT-3’ |
| EcRA forward | 5’-CCGTTACGGTTTACGCTAGTG-3’ |
| EcRA reverse | 5’-TAGCCATTGGACATGGTGGTA-3’ |
| EcRB forward | 5’-GCAGGGTTGACATCAAGTATC-3’ |
| EcRB reverse | 5’-CTAATCGCACGTTCTGCTTCA-3’ |
| USPA forward | 5’-TACGTGACAACGTGTCCGAGCG-3’ |
| USPA reverse | 5’-TGTATTCGTACCGCGTCCGTCG-3’ |
| USPB forward | 5’-GCGGACGCCATGTTCGAATG-3’ |
| USPB reverse | 5’-GACCGGTCTTCACACATTGAGC-3’ |
| HR3 forward | 5’-GGCGTCCAATTACGGTGTGATA-3’ |
| HR3 reverse | 5’-ATTTGACCGCGTCGCGACTCAT-3’ |
| FTZ-F1A forward | 5’-AGCCCATCGAGATTAAGATTCCA-3’ |
| FTZ-F1A reverse | 5’-GCTCAATGGCGGTAGTACCTGA-3’ |
| FTZ-F1B forward | 5’-GCCAACAGCTACTCGCTCTACAA-3’ |
| FTZ-F1B reverse | 5’-GCTCAATGGCGGTAGTACCTGA-3’ |
| E74B forward | 5’-ACCGCCGACCGCAATACGAT-3’ |
| E74B reverse | 5’-AGCTGTCGACATCCGAAACAC-3’ |
| LK6 forward | 5’-AGTACAACCCCAACAGCAGCAA-3’ |
| LK6 reverse | 5’-TCAGTATCGGCACCACGGTATC-3’ |
| ATG8 forward | 5’-TAACCGTCGGCCAGTTCTAT-3’ |
| ATG8 reverse | 5’-GTAGCCGATGTTGGTGGAAT-3’ |
| DEBCL forward | 5’-CCGACGTCATTGAGGAAGAT-3’ |
| DEBCL reverse | 5’-AATGTAATTTCGGTCTGCGG-3’ |

Primers used to synthetize dsRNA to knockdown HR3 (This needs to be added to mat&methods)

5’-TAATACGACTCACTATAGGGATCGAGTTCGCCAAGCTGATA-3’

5’-TAATACGACTCACTATAGGGATCGAGAAGAGCTCCTTGTA-3’

TACAAGGAGCTCTTCTCGAT

(underlined is HR3 sequence. 5’ end (not underlined) is T7 sequence used to for synthesis of dsRNA.

This primers give a 545bp amplicon targeting the hinge/LBD region of HR3
